# Does the pattern of lymphatic drainage influence the risk of nodal recurrence in trunk melanoma patients with negative sentinel lymph node biopsy?^☆^^☆☆^

**DOI:** 10.1016/j.abd.2021.05.005

**Published:** 2021-10-05

**Authors:** Francisca Jácome Morgado, Paula Soeiro, Ana Brinca, André Pinho, Ricardo Vieira

**Affiliations:** a Department of Dermatovenerology, Coimbra University Hospital Centre, Coimbra, Portugal; b Department of Nuclear Medicine, Coimbra University Hospital Centre, Coimbra, Portugal; c Faculty of Medicine, University of Coimbra, Coimbra, Portugal

**Keywords:** Melanoma, Sentinel lymph node biopsy, Skin cancer

## Abstract

**Background:**

There are conflicting data regarding the prognostic value of the lymphatic basin drainage pattern in melanoma patients and the evidence is scant in the setting of negative sentinel lymph node biopsy.

**Objective:**

To investigate whether the pattern of lymphatic basin drainage influences the risk of nodal disease in patients with melanoma of the trunk and negative sentinel lymph node biopsy.

**Methods:**

A case series of patients with trunk melanoma and negative sentinel lymph node biopsy was retrospectively evaluated. Clinicopathological features, the pattern of lymphatic drainage and nodal, metastatic, and overall recurrence-free survival were reviewed.

**Results:**

Of the 135 patients included, multiple lymphatic basin drainage was identified in 61 (45.2%). Ten of the 74 (13.5%) patients with single drainage developed nodal recurrence, compared with 2 of the 61 (3.6%) patients with multiple drainages (p = 0.04). Nodal recurrence-free survival was significantly longer in the group with multiple drainages than in the group with single drainage (175.6 vs. 138.7 months; p = 0.04). In multivariate analysis, single drainage was associated with a higher risk of nodal recurrence (HR = 4.54; p = 0.05). No significant differences in metastatic and overall recurrence-free survival were found between groups.

**Study limitations:**

Retrospective analysis, single-center study, small sample, detailed histopathologic information not always present.

**Conclusions:**

In patients with trunk melanoma and negative sentinel lymph node biopsy, multiple lymphatic basin drainage may be an independent risk factor for nodal disease recurrence. This factor may help to identify patients with negative sentinel lymph node biopsy with a higher risk of nodal recurrence.

## Introduction

Primary cutaneous melanoma represents 4% of all skin cancers and is responsible for the majority of skin cancer-related deaths. Early diagnosis and effective treatment, at a stage where cure is still achievable, are primary endpoints.^1^

For early-stage melanoma, the strongest prognostic factors are Breslow thickness, ulceration, and Sentinel Lymph Node (SLN) status.^2^ The SLN is defined as the first lymph node(s) to which melanoma cells are most likely to spread, from the primary tumor. Sentinel Lymph Node Biopsy (SLNB) is a surgical procedure in which the SLN is examined, allowing the prediction of the nodal metastatic status of the patient.^3^, ^4^ This technique is preceded by a preoperative lymphoscintigraphy to identify the draining nodal basin(s), which is particularly important for regions with highly variable drainage such as the trunk, head and neck.^5^

Currently, instead of identifying candidates for complete lymph node dissection, the SLNB plays an important role in the appropriate staging of the patients and the selection of candidates for adjuvant therapy.^6^, ^7^

Multiple lymphatic basin drainage (multiple-LBD) occurs when the primary tumor drains to multiple nodal basins and it occurs more frequently in patients with melanoma of the trunk (17%–46%) than in other locations, such as limbs or head and neck.^8^, ^9^ Whether multiple-LBD or single-LBD have a prognostic value is under evaluation. Studies regarding this issue report contradictory findings and little is known in the case of patients with negative SLNB.^10^, ^11^, ^12^, ^13^

We aimed to investigate whether the pattern of lymphatic basin drainage influences the risk of nodal disease in patients with melanoma of the trunk and negative SLNB. As a secondary goal, we aimed to assess the false-negative rate of SLBN in trunk melanomas.

## Methods

The research was conducted according to the local Ethical Committee.

### Patients

We performed an observational retrospective study including all patients with cutaneous melanoma of the trunk with negative SLNB, diagnosed between October 2004 and September 2017 at Coimbra University Hospital Centre, Coimbra, Portugal. Medical records were analyzed from the first dermatologic consultation until the last evaluation or death. Patients with at least 12 months of follow-up, or less, in case of earlier progression to nodal or metastatic disease were included.

Two groups were considered for comparison: patients with multiple-LBD and single-LBD. Patients were considered to have multiple-LBD when SLNB was performed in more than one regional lymph node basin, regardless of the total number of nodes excised.

The endpoints included the first nodal or metastatic disease after negative SLNB in a patient not submitted to any other medical or surgical treatment so far.

Patients’ clinical features (age, gender and tumor localization on the trunk), melanoma histological features (Breslow depth, ulceration, and histological subtype), the pattern of lymphatic drainage (number and localization of node basins explored and sentinel lymph nodes excised), nodal recurrence-free survival (nodal-RFS), metastatic recurrence-free survival (metastatic-RFS) and overall recurrence-free survival (overall-RFS) were reviewed.

Since the study was conducted within a 14-year time period, differences in the approach to the regional and distant metastatic disease (namely different indications for complete lymph node dissection, or metastatic disease surgery; indications for systemic therapy of metastatic disease with chemotherapy, targeted therapy or immune checkpoint inhibitor therapy; and more recently adjuvant therapy) were registered.

However, for the purpose of this study, these changes in the guidelines could not impact the present study’s results since they did not influence the drainage pattern and only recurrence-free survivals (i.e., before the effect of any possible complete lymph node dissection, or systemic therapy either for metastatic disease or in the adjuvant setting) were considered.

### Primary excision, preoperative lymphoscintigraphy, and SLN technique

The diagnosis of melanoma was based upon excisional biopsy or incisional biopsy followed by radical excision and histopathology analysis. An excisional biopsy was performed with narrow margins. Wide local excision with a margin of 1 or 2 cm depending on Breslow thickness.

Patients were selected for SLNB according to the recommended guidelines at that time.

A 2-day protocol using [^99m^Tc] Tc-human serum albumin nanocolloid was used to perform a SLN scintigraphy: On day-1, the injections were made at the primary melanoma site. Immediately after the injection a dynamic imaging study with the field of view centered on the lesion was started. Static images 20-minutes and 2-hours after injection were acquired. Two and half hours after tracer injection, a SPECT with low-dose CT scan without contrast was made. On day 2 SLN biopsy was attempted on all nodal basins that had been identified by preoperative lymphoscintigraphy. For this, a portable gamma-probe was used intraoperatively, to detect the emission of radiation from SLN, thus allowing its identification and excision.

Nodal basins included right and left axilla, neck, groin, scapular, supraclavicular, intercostal, or submandibular. In-transit or satellite nodes were not considered as a separate basin.

During the study period, the lymphoscintigraphy protocol remain unchanged and was performed by the same nuclear medicine team. The SLNB procedure was also accomplished by the same dermatologic surgical team of the hospital.

### Statistical methods

IBM SPSS® software, version 25.0 (Armonk, NY: IBM Corp.) was used. Only p-values of non-parametric tests were reported since most continuous variables had a non-normal distribution. The Chi-Square test was used to compare categorical variables between multiple and single-LBD groups. Kaplan-Meier method and log-rank test were used to compare survival estimates between groups. Logistic regression was used to test for the significance of the predictor variables. Statistical significance was set at p-value ≤0.05.

## Results

### Clinico-pathologic characteristics

During the study period, 206 patients had a diagnosis of melanoma of the trunk and were selected to perform SLNB in the present study’s institution. Of these, 147 had negative SLNB and were eligible for the present study. The patients who were lacking clinical or pathological information or did not fulfill the required follow-up time were excluded and a total of 135 patients were included.

The clinical characteristics of the patients and pathological features of primary melanoma, according to the nodal drainage pattern, are summarized in Table 1.

**Table 1:** Clinical and pathological characteristics of the study cohort and distribution according to nodal basin drainage.

	Single-LBD	Multiple-LBD	Total	p
Patients	74	61	135	
Gender				0.66
Female	30	27	57	
Male	44	34	78	
Age (median; min.–max.)	71 (35–95)	68 (33–87)	69 (33–95)	0.53
Breslow (median; min–max.)	1.72 (0.5–15)	1.50 (0.8–15)	1.7 (0.5–15)	0.69
T				0.32
T1	13	15	28	
T2	31	22	52	
T3	15	17	32	
T4	15	7	22	
Ulceration				0.17
Yes	22	25	47	
No	52	36	88	
Histologic subtype				0.12
Superficial spreading	34	26	70	
Nodular	19	20	39	
Nevoid	0	1	1	
Spitzoid	0	1	1	
Desmoplastic	0	1	1	
Other^b^	7	0	7	
Unknown^c^	14	12	26	
Localization				0.47
Upper trunk	59	42	81	
Lower trunk	15	19	34	
Localization				0.01^a^
Anterior	24 (75.0%)	8 (25.0%)	32	
Posterior	50 (48.5%)	53 (51.5%)	103	
Any nodal (regional) recurrence	10 (83.3%)	2 (16.7%)	12	0.04^a^
Any metastatic (distant) recurrence	21 (63.6%)	12 (36.3%)	33	0.23
Any (overall) disease recurrence	24 (66.7%)	12 (33.3%)	36	0.09

Single-LBD, Single Lymph node Basin Drainage, Multiple-LBD, Multiple Lymph Node Basin Drainage.

a Statistically significant difference.

b Other histologic subtypes included: animal-type melanoma, intradermal melanoma, melanoma blue nevus, melanoma in congenital melanocytic naevi.

c Cases missing histologic subtype classification, but fulfilling other variables studied.

Seventy-eight patients (57.8%) were males, with a median age of 69 years (33–95). Melanoma was located in the upper trunk in 101 patients (74.8%), in the inferior trunk in 34 (25.2%), in the front in 32 (23.7%), and in the back in 103 (76.3%).

Histologic subtypes of melanoma identified were superficial spreading (60), nodular (39), nevoid (1), spitzoid (1), desmoplastic (1), or other (7). In 26 cases the histologic subtype classification was missing, despite having information about all other studied variables.

Median Breslow depth was 1.7 mm and ulceration was present in 47 (34.8%) patients.

During the follow-up period, 3 patients (2%) had an only nodal recurrence, 24 (17.8%) developed exclusively metastatic recurrence and 9 (6.7%) had both nodal and metastatic recurrence.

Multiple-LBD was identified in 61 (45.2%) patients, and in most of them (88.5%) only 2 basins were explored. In 35 (57.3%) patients with multiple-LBD, there was bilateral symmetric identification of SLN, being both axilla in 30 (46.6%) patients and both groins in 5 (8.2%). In 6 patients, 3 basins were explored, and in a single patient, drainage occurred to 4 basins.

Of the 74 patients with single-LBD, the SLN was located mostly in the axilla (63), and less often on the groin (7), cervical area (1), or other uncommon basins (3) (scapular and supraclavicular) (Table 2).

**Table 2:** Anatomic distribution of single and multiple lymphatic basin drainage.

Single-LBD (n = 74)		Multiple-LBD (n = 61)	Nº of basins		
			2	3	4
Axilla	63	Axilla, bilateral	30	–	–
Groin	7	Groin, bilateral	5	–	–
Cervical	1	Axilla + groin	5	2	1
Uncommon^a^	3	Axilla + neck	3	–	–
		Axilla + uncommon^a^	7	4	–
		Neck + uncommon^a^	2	–	–
		Multiple uncommon^a^	2	–	–

Single-LBD, Single Lymph node Basin Drainage; Multiple-LBD, Multiple Lymph node Basin Drainage.

a Include scapular, supraclavicular, intercostal, or submandibular.

Posterior trunk melanomas were more often associated with multiple-LBD (51.5%), while in anterior trunk melanomas single-LBD was more common (75%), being these differences statistically significant.

Any other clinical or histological feature significantly associated with the drainage pattern (multiple-LBD and single-LBD).

### Lymphatic basin drainage, relapse-free survival (RFS) and risk of disease

The median follow-up period was 5.6 years (range 1–15 years).

**Figure 1:**
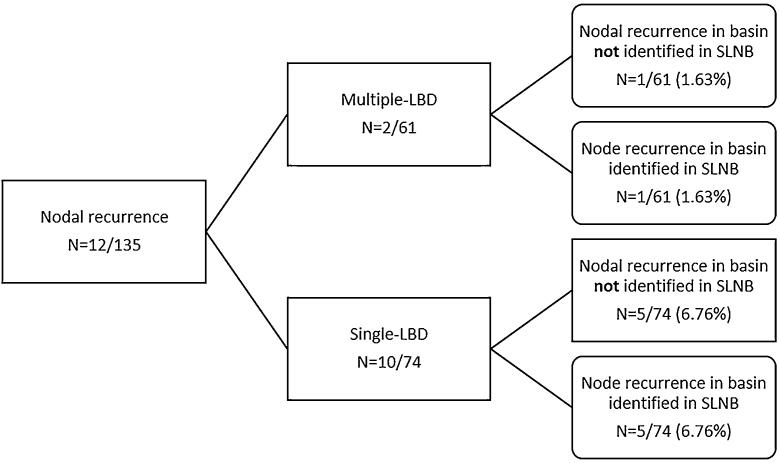
Lymph node basin drainage of patients with nodal recurrence. Single-LBD, Single Lymph node Basin Drainage; Multiple-LBD, Multiple Lymph node Basin Drainage.

Ten of the 74 (13.5%) patients with single-LBD developed nodal recurrence compared with 2 of the 61 (3.6%) patients with multiple-LBD (p = 0.04) (Fig. 1 and Table 1).

In the single-LBD group, 5 patients had a nodal recurrence in the same basin identified in SLNB, meaning a false negative rate of SLNB of 6.76% (5/74). In another 5 patients with single-LBD, nodal recurrence developed in a regional basin not identified in SLNB.

Among the 2 patients with multiple-LBD, only 1 developed nodal recurrence in a territory not previously studied by SLNB, meaning a false negative rate of 1.63% (1/61). The overall false-negative rate for SLNB was 4.4% (6/135).

**Figure 2:**
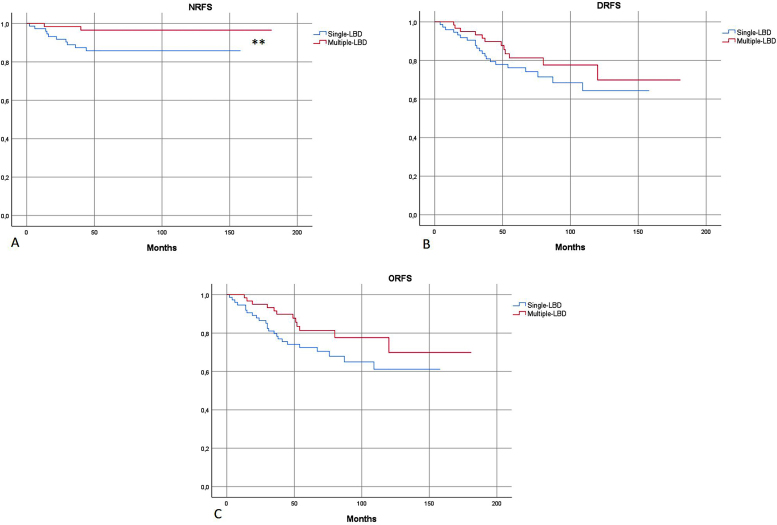
(A) Kaplan-Meier: Nodal Recurrence-Free Survival (NRFS) was significantly more favorable in patients with multiple-LBD. (B and C) No significant differences in Distant Recurrence-Free Survival (DRFS) and Overall Recurrence-Free Survival (ORFS) were found, according to drainage pattern. Single-LBD, Single Lymphatic Basin Drainage; Multiple-LBD, Multiple Lymphatic Basin Drainage. ** Statistically significant difference in survival curves.

In survival analysis, nodal-RFS was significantly longer in the group with multiple-LBD, than in the group with single-LBD (175.6 vs. 138.7 months; p = 0.04) (Fig. 2).

On univariate analysis, single-LBD (HR = 4.34; p = 0.05) and Breslow depth (HR = 1.36; p = 0.05) were the only factors significantly associated with a higher risk of nodal recurrence, in negative-SLNB patients.

After adjustment for Breslow depth, the single-LBD remained associated with a higher risk of nodal recurrence (HR = 4.54; p = 0.05) (Table 3).

**Table 3:** Risk Factors for Nodal recurrence.

Nodal disease risk factors	HR	p
Univariable Cox regression		
Age	1.05	0.06
Male	1.50	0.50
Localization: upper trunk	0.03	0.21
Localization: anterior trunk	0.93	0.94
Breslow	1.36	0.00^a^
Ulceration	2.90	0.06
Single-LBD	4.34	0.05^a^
Number of basins	0.24	0.06
Number of SN	0.75	0.32
Multivariable Cox regression		
Single-LBD & Breslow	4.54	0.05

Single-LBD, Single Lymph node Basin Drainage; SN, Sentinel lymph Node; HR, Hazard Ratio.

a Statistically significant difference.

Although not statically significant, there was a tendency for a lower risk of nodal disease when the number of basins increased (HR = 0.24; p = 0.06).

The number of lymph nodes excised did not correlate with nodal disease risk (HR = 0.75; p = 0.32).

No significant differences in metastatic-RFS and overall-RFS were found between single or multiple-LDB patients (Fig. 2). On logistic regression, the patter of lymphatic drainage did not affect the risk of metastatic (distant) or overall (nodal and metastatic) disease recurrency (p = 0.27 and p = 0.12, respectively).

## Discussion

Several studies have evaluated whether multiple-LBD or single-LBD have a prognostic value, concerning nodal or metastatic disease in trunk melanoma patients.^6^, ^10^, ^11^, ^12^, ^13^, ^14^, ^15^, ^16^ However, the conclusions are divergent which can be related to the inclusion of both positive and negative SLNB patients. Indeed, it is well known that positive SLNB is a strong prognostic factor, and for an accurate evaluation, those groups should be assessed independently. Only a few researchers analyzed the impact of the drainage pattern in negative SLNB patients.^8^, ^14^, ^15^

Given the controversial results, we retrospectively evaluated a case series of trunk melanomas with negative SLNB. Melanomas of the trunk were choiced since they are responsible for the majority of multiple-LBD and we selected a sole anatomical area to gather a more homogeneous cohort of patients.

We could observe that the risk of nodal (regional) disease was 4.54 times higher in the single-LBD than in multiple-LBD group, while no difference in metastatic or overall (nodal and metastatic) disease was found between groups.

Ribero et al. in a single-center study (2013), found that in negative SLNB patients with trunk melanoma, single-LBD was associated with a higher percentage of disease recurrence compared to multiple-LBD (19% vs. 7%, p = 0.03).^8^ However, the authors did not provide information on nodal and metastatic disease recurrence, separately.

Later, in a multicenter study (2016), Ribero et al. found that, in negative SLNB patients, multiple-LBD was associated with a lower risk of melanoma recurrence (HR = 0.73, p = 0.05) and melanoma-related death (HR = 0.68, p = 0.001), although no difference in nodal progression between groups was found.^14^

On the contrary, Pinero et al. showed that in negative SLNB patients, disease (nodal and metastatic) free survival was poor in multiple-LBD but included melanomas of the trunk and limbs.^15^ Investigating melanoma from different anatomic areas, that could have different genetic prognoses, could yield risk of bias.

Jimenez et al. reported that negative SLNB patients with trunk melanoma and multiple-LBD had a less favorable recurrence-free survival (76 months vs. 190 months for single-LBD, p = 0.06) and overall survival (76 months vs. 190 months for single-LBD, p = 0.04). This study has the limitation of a shorter median follow-up (27 months).^11^

With a median follow-up time of more than 5 years, the present study’s data suggest the concept that in trunk melanomas of negative SLNB patients, multiple-LBD is associated with an increased nodal recurrence-free interval, while no difference in metastatic or overall (metastatic and nodal) disease-free intervals was found, according to the drainage pattern.

We hypothesized that in patients with multiple-LBD, a negative SLNB might better reflect a true absence of nodal disease i.e., the negative predictive value of SLNB might be higher, once more basins were evaluated and absence of disease was documented. In the present study’s sample, the development of nodal disease in a territory not previously studied by SLNB, in half of the cases with single-LBD, may corroborate this hypothesis, underlining a possible underestimation of other lymphatic pathways with the lymphoscintigraphy.

A possibility is that single-LBD may occur due to the obstruction of lymphatic channels by melanoma cells, not allowing recognition of migratory routes in the lymphoscintigraphy, ultimately leading to negative SLNB. Although the Breslow depth was not significantly different in both groups, the lymphovascular invasion or embolization was not assessed, being not possible to rule out if this factor was the cause of such difference.

One might call into question the accuracy of the original lymphoscintigraphy, but we found an overall false-negative rate around 4%, similar to, or even lower than values reported in other series (4%–10.8%).^8^, ^17^

Limitations of this study include its single-center nature, the small sample size, and the retrospective analysis. We did not evaluate overall survival, because the study was conducted within a period of 14 years, thus including patients treated differently; with the recent introduction of targeted therapies and immunotherapy, there was a dramatic improvement in survival compared with former patients treated with chemotherapy. If we had compared overall survival between groups, it would lead to treatment bias. Additionally, the histopathological assessment of the presence of lymphovascular invasion would enrich the present study, to corroborate with our hypothesis.

## Conclusion

The presence of multiple-LBD is common in trunk melanoma patients, and in patients with negative SLNB, it may be an independent risk factor for nodal disease recurrence. Although the drainage pattern does not seem to influence the overall-recurrence-free survival, this factor may help to identify patients with negative SLNB with a higher risk of nodal recurrence, including in basins not previously studied by SLNB.

## Financial support

None declared.

## Authors’ contributions

Francisca Morgado: Study conception and planning; critical literature review; data collection, analysis, and interpretation; preparation and writing of the manuscript.

Paula Soeiro: Data collection; analysis; interpretation.

Ana Brinca: Intellectual participation in propaedeutic and/or therapeutic management of studied cases.

André Pinho: Statistical analysis; study conception and planning; intellectual participation in propaedeutic and/or therapeutic management of studied cases; effective participation in research orientation; manuscript critical review.

Ricardo Vieira: Study conception and planning; intellectual participation in propaedeutic and/or therapeutic management of studied cases; approval of the final version of the manuscript; effective participation in research orientation; manuscript critical review.

## Conflicts of interest

None declared.
